# Aqua­bis(4-formyl­benzoato-κ^2^ *O* ^1^,*O* ^1′^)bis­(isonicotinamide-κ*N* ^1^)cadmium(II) monohydrate

**DOI:** 10.1107/S1600536809042640

**Published:** 2009-10-23

**Authors:** Tuncer Hökelek, Filiz Yılmaz, Barış Tercan, Ferdi Gürgen, Hacali Necefoğlu

**Affiliations:** a Department of Physics, Hacettepe University, 06800 Beytepe, Ankara, Turkey; b Department of Chemistry, Faculty of Science, Anadolu University, 26470 Yenibağlar, Eskişehir, Turkey; c Department of Physics, Karabük University, 78050 Karabük, Turkey; d Department of Chemistry, Kafkas University, 63100 Kars, Turkey

## Abstract

The asymmetric unit of the title Cd^II^ complex, [Cd(C_8_H_5_O_3_)_2_(C_6_H_6_N_2_O)_2_(H_2_O)]·H_2_O, contains two 4-formyl­benzoate (FB), two isonicotinamide (INA) ligands, one coordinated and one uncoordinated water mol­ecule; the FB ions act as bidentate ligands. The coordination number of the Cd(II) atom is seven within a CdO_5_N_2_ donor set. Intra­molecular O—H⋯O hydrogen bonds link the uncoordinated water mol­ecules to the carboxyl groups. The dihedral angle between the carboxyl­ate groups and the adjacent benzene rings are 17.53 (13) and 16.55 (14)°. In the crystal structure, inter­molecular O—H⋯O, N—H⋯O, N—H⋯N and C—H⋯O hydrogen bonds link the mol­ecules into a supra­molecular structure. The amide group of one of the INA ligands is disordered over two orientations, with an occupancy ratio of 0.759 (3):0.241 (3).

## Related literature

For niacin, see: Krishnamachari (1974 ▶) and for the nicotinic acid derivative *N*,*N*-diethyl­nicotinamide, see: Bigoli *et al.* (1972 ▶). For related structures, see: Greenaway *et al.* (1984 ▶); Hökelek & Necefoğlu (1996 ▶); Hökelek *et al.* (2009 ▶).
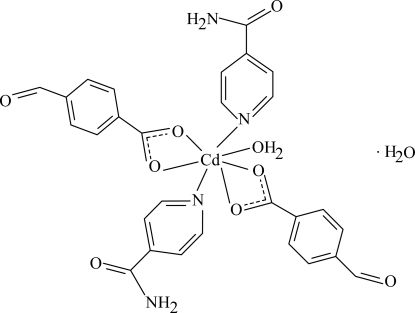


## Experimental

### 

#### Crystal data

[Cd(C_8_H_5_O_3_)_2_(C_6_H_6_N_2_O)_2_(H_2_O)]·H_2_O
*M* *_r_* = 690.93
Monoclinic, 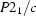

*a* = 9.3357 (3) Å
*b* = 19.0501 (6) Å
*c* = 16.3743 (5) Å
β = 93.203 (1)°
*V* = 2907.55 (16) Å^3^
*Z* = 4
Mo *K*α radiation
μ = 0.82 mm^−1^
*T* = 100 K
0.27 × 0.11 × 0.10 mm

#### Data collection

Bruker Kappa APEXII CCD area-detector diffractometer
Absorption correction: multi-scan (*SADABS*; Bruker, 2005 ▶) *T*_min_ = 0.896, *T*_max_ = 0.920
26549 measured reflections
7151 independent reflections
5904 reflections with *I* > 2σ(*I*)
*R*_int_ = 0.043

#### Refinement

*R*[*F* ^2^ > 2σ(*F* ^2^)] = 0.028
*wR*(*F* ^2^) = 0.075
*S* = 1.03
7151 reflections
431 parameters
8 restraints
H atoms treated by a mixture of independent and constrained refinement
Δρ_max_ = 1.16 e Å^−3^
Δρ_min_ = −0.63 e Å^−3^

### 

Data collection: *APEX2* (Bruker, 2007 ▶); cell refinement: *SAINT* (Bruker, 2007 ▶); data reduction: *SAINT*; program(s) used to solve structure: *SHELXS97* (Sheldrick, 2008 ▶); program(s) used to refine structure: *SHELXL97* (Sheldrick, 2008 ▶); molecular graphics: *ORTEP-3 for Windows* (Farrugia, 1997 ▶); software used to prepare material for publication: *WinGX* (Farrugia, 1999 ▶).

## Supplementary Material

Crystal structure: contains datablocks I, global. DOI: 10.1107/S1600536809042640/xu2635sup1.cif

Structure factors: contains datablocks I. DOI: 10.1107/S1600536809042640/xu2635Isup2.hkl

Additional supplementary materials:  crystallographic information; 3D view; checkCIF report

## Figures and Tables

**Table 1:** Selected bond lengths (Å)

Cd1—O1	2.6055 (13)
Cd1—O2	2.3066 (13)
Cd1—O3	2.3368 (13)
Cd1—O4	2.5117 (13)
Cd1—O9	2.3271 (15)
Cd1—N1	2.3200 (16)
Cd1—N3	2.3362 (16)

**Table 2:** Hydrogen-bond geometry (Å, °)

*D*—H⋯*A*	*D*—H	H⋯*A*	*D*⋯*A*	*D*—H⋯*A*
N2—H2*A*⋯O7^i^	0.86	2.04	2.891 (2)	172
N2—H2*B*⋯O10^ii^	0.86	2.05	2.888 (3)	165
N4—H4*A*⋯O8^iii^	0.86	2.01	2.863 (3)	174
N4—H4*B*⋯O5^iv^	0.86	2.01	2.852 (3)	166
N4—H4*A*⋯N4*B*^iii^	0.86	2.35	3.096 (7)	146
N4*B*—H4*B*1⋯N4^iii^	0.86	2.35	3.096 (7)	145
N4*B*—H4*B*1⋯O8*B*^iii^	0.86	2.02	2.880 (9)	178
N4*B*—H4*B*2⋯O7^v^	0.86	2.12	2.941 (7)	158
O9—H91⋯O4^vi^	0.884 (15)	1.86 (2)	2.740 (2)	175 (2)
O9—H92⋯O1^v^	0.875 (16)	1.87 (2)	2.740 (2)	174 (2)
O10—H101⋯O3	0.880 (16)	2.32 (3)	2.876 (2)	122 (2)
O10—H102⋯O2	0.864 (18)	1.90 (3)	2.766 (2)	175 (3)
C11—H11⋯O8^ii^	0.93	2.55	3.315 (3)	140
C17—H17⋯O1^v^	0.93	2.44	3.212 (2)	140
C20—H20⋯O10^ii^	0.93	2.35	3.258 (3)	166
C23—H23⋯O4^vi^	0.93	2.48	3.253 (2)	140
C26—H26⋯O6^vii^	0.93	2.55	3.137 (3)	122
C27—H27⋯O6^vii^	0.93	2.51	3.133 (3)	124

Symmetry codes: (i) 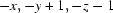; (ii) 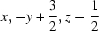; (iii) 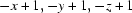; (iv) 
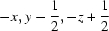
; (v) 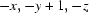; (vi) 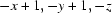; (vii) 
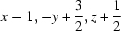
.

**Fig. 1.**
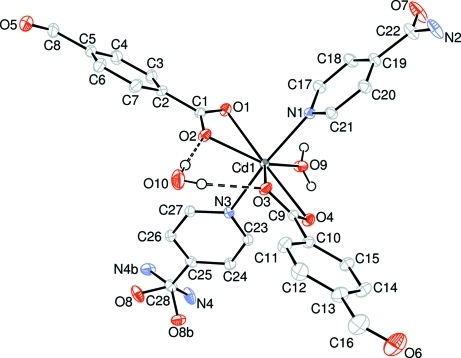
The molecular structure of the title molecule with the atom-numbering scheme. Displacement ellipsoids are drawn at the 50% probability level. Dashed lines indicate the hydrogen-bondings. Hydrogen atoms except of water molecules have been omitted for clarity.

## supplementary crystallographic information

### Comment

As a part of our ongoing investigation on transition metal complexes of nicotinamide (NA), one form of niacin (Krishnamachari, 1974), and/or the nicotinic acid derivative *N*,*N*-diethylnicotinamide (DENA), an important respiratory stimulant (Bigoli *et al.*, 1972), the title compound was synthesized and its crystal structure is reported herein.

In the monomeric title complex, (I), the Cd^II^ ion is surrounded by two formylbenzoate (FB) and two isonicotinamide (INA) ligands and one water molecule. The FB ions act as bidentate ligands, while the INA ions are monodentate ligands. The structures of similar complexes of Zn^II^ ion, [Zn_2_(C_10_H_14_N_2_O)_2_(C_7_H_5_O_3_)_4_].2H_2_O, (II) (Hökelek & Necefoğlu, 1996) and [Zn(C_9_H_10_NO_2_)_2_(C_6_H_6_N_2_O)(H_2_O)_2_], (III) (Hökelek *et al.*, 2009) have also been determined.

In the title compound (Fig. 1), the average Cd—O bond length (Table 1) is 2.4175 (13) Å and the Cd atom is displaced out of the least-squares planes of the carboxylate groups (O1/C1/O2) and (O3/C9/O4) by 0.1556 (2) Å and -0.0577 (2) Å, respectively. The dihedral angle between the planar carboxylate groups and the adjacent benzene rings A (C2—C7) and B (C10—C15) are 17.53 (13)° and 16.55 (14)°, respectively, while those between rings A, B, C (N1/C17—C21) and D (N3/C23—C27) are A/B = 7.05 (6), A/C = 69.34 (6), A/D = 66.49 (6), B/C = 68.78 (6), B/D = 73.54 (6) and C/D = 87.43 (5) °. The two four-membered rings, (Cd1/O1/O2/C1) and (Cd1/O3/O4/C9), are oriented at a dihedral angle of 20.06 (6)°. The intramolecular O—H···O hydrogen bonds (Table 2) link the uncoordinated water molecule to the carboxylate groups. In (I), the O1—Cd1—O2 and O3—Cd1—O4 angles are 52.91 (4) and 53.96 (4) °, respectively. The corresponding O—M—O (where *M* is a metal) angles are 58.3 (3)° in (II), 60.03 (6)° in (III) and 55.2 (1)° in [Cu(Asp)_2_(py)_2_] (where Asp is acetylsalicylate and py is pyridine) [(IV); Greenaway *et al.*, 1984].

In the crystal structure, intramolecular O—H···O and intermolecular O—H···O, N—H···O, N—H···N and C—H···O hydrogen bonds (Table 2) link the molecules into a supramolecular structure, in which they may be effective in the stabilization of the structure.

### Experimental

The title compound was prepared by the reaction of 3CdSO_4_.8H_2_O (3.85 g, 5 mmol) in H_2_O (25 ml) and INA (1.22 g, 10 mmol) in H_2_O (40 ml) with sodium 4-formylbenzoate (1.72 g, 10 mmol) in H_2_O (50 ml). The mixture was filtered and set aside to crystallize at ambient temperature for several days, giving colorless single crystals.

### Refinement

Atoms H8 and H16 (for methine) and H91, H92, H101 and H102 (for H_2_O) were located in difference Fourier map and refined isotropically, with restrains of O9—H91 = 0.894 (15), O9—H92 = 0.875 (16), O10—H101 = 0.880 (16) and O10—H102 = 0.864 (18) Å and H91—O9—H92 = 106 (2) and H101—O10—H102 = 106 (3) °. The remaining H atoms were positioned geometrically with N—H = 0.86 Å (for NH_2_) and C—H = 0.93 Å for aromatic H atoms and constrained to ride on their parent atoms, with *U*_iso_(H) = 1.2*U*_eq_(C,*N*). The O8, N4, H4A and H4B atoms are disordered over two orientations. During the refinement process, the disordered O8, N4, H4A, H4B and O8B, N4B, H4B1, H4B2 were refined with occupancies of 0.759 (3) and 0.241 (3), respectively.

### Crystal data

**Table:**

[Cd(C_8_H_5_O_3_)_2_(C_6_H_6_N_2_O)_2_(H_2_O)]·H_2_O	*F*(000) = 1400
*M**_r_* = 690.93	*D*_x_ = 1.578 Mg m^−^^3^
Monoclinic, *P*2_1_/*c*	Mo *K*α radiation, λ = 0.71073 Å
Hall symbol: -P 2ybc	Cell parameters from 9959 reflections
*a* = 9.3357 (3) Å	θ = 2.4–28.4°
*b* = 19.0501 (6) Å	µ = 0.82 mm^−^^1^
*c* = 16.3743 (5) Å	*T* = 100 K
β = 93.203 (1)°	Block, colorless
*V* = 2907.55 (16) Å^3^	0.27 × 0.11 × 0.10 mm
*Z* = 4	

### Data collection

**Table:**

Bruker Kappa APEXII CCD area-detector diffractometer	7151 independent reflections
Radiation source: fine-focus sealed tube	5904 reflections with *I* > 2σ(*I*)
graphite	*R*_int_ = 0.043
φ and ω scans	θ_max_ = 28.4°, θ_min_ = 1.6°
Absorption correction: multi-scan (*SADABS*; Bruker, 2005)	*h* = −12→12
*T*_min_ = 0.896, *T*_max_ = 0.920	*k* = −20→25
26549 measured reflections	*l* = −21→17

### Refinement

**Table:**

Refinement on *F*^2^	Primary atom site location: structure-invariant direct methods
Least-squares matrix: full	Secondary atom site location: difference Fourier map
*R*[*F*^2^ > 2σ(*F*^2^)] = 0.028	Hydrogen site location: inferred from neighbouring sites
*wR*(*F*^2^) = 0.075	H atoms treated by a mixture of independent and constrained refinement
*S* = 1.03	*w* = 1/[σ^2^(*F*_o_^2^) + (0.0355*P*)^2^ + 1.7245*P*] where *P* = (*F*_o_^2^ + 2*F*_c_^2^)/3
7151 reflections	(Δ/σ)_max_ = 0.002
431 parameters	Δρ_max_ = 1.16 e Å^−^^3^
8 restraints	Δρ_min_ = −0.63 e Å^−^^3^

### Special details

**Table:**

Geometry. All e.s.d.'s (except the e.s.d. in the dihedral angle between two l.s. planes) are estimated using the full covariance matrix. The cell e.s.d.'s are taken into account individually in the estimation of e.s.d.'s in distances, angles and torsion angles; correlations between e.s.d.'s in cell parameters are only used when they are defined by crystal symmetry. An approximate (isotropic) treatment of cell e.s.d.'s is used for estimating e.s.d.'s involving l.s. planes.
Refinement. Refinement of *F*^2^ against ALL reflections. The weighted *R*-factor *wR* and goodness of fit *S* are based on *F*^2^, conventional *R*-factors *R* are based on *F*, with *F* set to zero for negative *F*^2^. The threshold expression of *F*^2^ > σ(*F*^2^) is used only for calculating *R*-factors(gt) *etc*. and is not relevant to the choice of reflections for refinement. *R*-factors based on *F*^2^ are statistically about twice as large as those based on *F*, and *R*- factors based on ALL data will be even larger.

### Fractional atomic coordinates and isotropic or equivalent isotropic displacement parameters (Å^2^)

**Table:**

	*x*	*y*	*z*	*U*_iso_*/*U*_eq_	Occ. (<1)
Cd1	0.254193 (11)	0.604747 (6)	−0.000741 (7)	0.01128 (5)	
N1	0.16048 (16)	0.59088 (8)	−0.13399 (10)	0.0149 (3)	
N2	0.1001 (2)	0.56817 (10)	−0.44328 (10)	0.0292 (4)	
H2A	0.0791	0.5559	−0.4930	0.035*	
H2B	0.1589	0.6022	−0.4330	0.035*	
N3	0.34349 (16)	0.58841 (8)	0.13406 (9)	0.0147 (3)	
N4	0.4990 (3)	0.46566 (12)	0.39673 (14)	0.0269 (6)	0.759 (3)
H4A	0.5191	0.4518	0.4460	0.032*	0.759 (3)
H4B	0.5033	0.4366	0.3567	0.032*	0.759 (3)
N4B	0.3548 (7)	0.5230 (4)	0.4293 (4)	0.0264 (19)	0.241 (3)
H4B1	0.3709	0.5103	0.4794	0.032*	0.241 (3)
H4B2	0.2682	0.5300	0.4103	0.032*	0.241 (3)
O1	−0.00985 (14)	0.59254 (7)	0.04111 (9)	0.0179 (3)	
O2	0.10880 (13)	0.69266 (7)	0.04495 (8)	0.0184 (3)	
O3	0.39326 (13)	0.69982 (7)	−0.03952 (8)	0.0182 (3)	
O4	0.51129 (14)	0.59950 (7)	−0.03809 (9)	0.0171 (3)	
O5	−0.48043 (16)	0.85486 (9)	0.22050 (10)	0.0368 (4)	
O6	1.08430 (17)	0.78213 (9)	−0.20603 (11)	0.0406 (4)	
O7	−0.04317 (19)	0.48524 (9)	−0.39285 (9)	0.0374 (4)	
O8	0.4524 (2)	0.57695 (10)	0.43632 (11)	0.0255 (5)	0.759 (3)
O8B	0.5920 (5)	0.5229 (3)	0.4041 (3)	0.0222 (15)	0.241 (3)
O9	0.25468 (13)	0.48260 (8)	−0.00288 (8)	0.0204 (3)	
H91	0.327 (2)	0.4540 (11)	0.0103 (16)	0.037 (7)*	
H92	0.179 (2)	0.4560 (12)	−0.0130 (17)	0.042 (8)*	
O10	0.28361 (18)	0.80970 (9)	0.06005 (11)	0.0374 (4)	
H101	0.336 (3)	0.8070 (17)	0.0170 (15)	0.075 (11)*	
H102	0.226 (3)	0.7741 (15)	0.058 (2)	0.100 (14)*	
C1	−0.00109 (18)	0.65688 (10)	0.05789 (11)	0.0152 (4)	
C2	−0.12241 (18)	0.69296 (10)	0.09768 (11)	0.0167 (4)	
C3	−0.22577 (19)	0.65346 (11)	0.13505 (12)	0.0215 (4)	
H3	−0.2209	0.6047	0.1349	0.026*	
C4	−0.3363 (2)	0.68729 (12)	0.17261 (13)	0.0262 (5)	
H4	−0.4053	0.6611	0.1979	0.031*	
C5	−0.3441 (2)	0.75969 (12)	0.17262 (13)	0.0255 (5)	
C6	−0.2407 (2)	0.79938 (12)	0.13549 (13)	0.0258 (5)	
H6	−0.2464	0.8481	0.1354	0.031*	
C7	−0.1293 (2)	0.76589 (11)	0.09865 (13)	0.0222 (4)	
H7	−0.0591	0.7922	0.0746	0.027*	
C8	−0.4633 (2)	0.79273 (13)	0.21423 (15)	0.0323 (5)	
H8	−0.539 (3)	0.7581 (15)	0.2423 (17)	0.053 (8)*	
C9	0.50454 (18)	0.66490 (10)	−0.05055 (11)	0.0142 (3)	
C10	0.63231 (18)	0.70288 (10)	−0.08114 (11)	0.0158 (4)	
C11	0.6381 (2)	0.77562 (10)	−0.07766 (13)	0.0216 (4)	
H11	0.5634	0.8007	−0.0563	0.026*	
C12	0.7556 (2)	0.81089 (11)	−0.10619 (13)	0.0257 (4)	
H12	0.7610	0.8595	−0.1025	0.031*	
C13	0.8650 (2)	0.77348 (11)	−0.14008 (13)	0.0228 (4)	
C14	0.85761 (19)	0.70082 (11)	−0.14571 (13)	0.0215 (4)	
H14	0.9300	0.6759	−0.1697	0.026*	
C15	0.74230 (19)	0.66558 (10)	−0.11555 (12)	0.0186 (4)	
H15	0.7382	0.6169	−0.1182	0.022*	
C16	0.9887 (2)	0.81047 (13)	−0.17276 (16)	0.0330 (5)	
H16	0.999 (3)	0.8554 (15)	−0.1612 (17)	0.049 (8)*	
C17	0.05844 (19)	0.54301 (10)	−0.15291 (12)	0.0179 (4)	
H17	0.0135	0.5210	−0.1106	0.021*	
C18	0.0173 (2)	0.52511 (10)	−0.23229 (12)	0.0200 (4)	
H18	−0.0532	0.4914	−0.2431	0.024*	
C19	0.0830 (2)	0.55820 (10)	−0.29624 (11)	0.0180 (4)	
C20	0.1849 (2)	0.60930 (10)	−0.27711 (12)	0.0190 (4)	
H20	0.2289	0.6334	−0.3183	0.023*	
C21	0.22006 (19)	0.62389 (10)	−0.19570 (11)	0.0168 (4)	
H21	0.2885	0.6583	−0.1833	0.020*	
C22	0.0421 (2)	0.53467 (11)	−0.38244 (12)	0.0250 (4)	
C23	0.44196 (19)	0.53843 (10)	0.15065 (11)	0.0168 (4)	
H23	0.4839	0.5165	0.1072	0.020*	
C24	0.4839 (2)	0.51813 (10)	0.22937 (12)	0.0188 (4)	
H24	0.5516	0.4828	0.2385	0.023*	
C25	0.42334 (19)	0.55129 (10)	0.29467 (11)	0.0162 (4)	
C26	0.3257 (2)	0.60491 (10)	0.27811 (12)	0.0179 (4)	
H26	0.2861	0.6293	0.3205	0.021*	
C27	0.28801 (19)	0.62164 (10)	0.19738 (11)	0.0165 (4)	
H27	0.2216	0.6573	0.1866	0.020*	
C28	0.4613 (2)	0.53171 (10)	0.38237 (11)	0.0196 (4)	

### Atomic displacement parameters (Å^2^)

**Table:**

	*U*^11^	*U*^22^	*U*^33^	*U*^12^	*U*^13^	*U*^23^
Cd1	0.01149 (8)	0.01184 (8)	0.01049 (8)	0.00038 (4)	0.00042 (5)	−0.00060 (5)
N1	0.0142 (7)	0.0165 (8)	0.0139 (8)	0.0007 (6)	0.0005 (6)	0.0003 (6)
N2	0.0474 (11)	0.0282 (10)	0.0115 (8)	−0.0121 (8)	−0.0024 (7)	−0.0003 (7)
N3	0.0147 (7)	0.0163 (8)	0.0130 (7)	0.0004 (6)	0.0006 (6)	−0.0012 (6)
N4	0.0490 (15)	0.0203 (12)	0.0109 (11)	0.0046 (10)	−0.0034 (10)	0.0018 (9)
N4B	0.020 (3)	0.048 (5)	0.011 (3)	−0.001 (3)	0.001 (3)	0.007 (3)
O1	0.0174 (6)	0.0163 (7)	0.0203 (7)	0.0021 (5)	0.0029 (5)	−0.0009 (5)
O2	0.0142 (6)	0.0177 (7)	0.0235 (7)	0.0011 (5)	0.0044 (5)	−0.0001 (6)
O3	0.0124 (6)	0.0183 (7)	0.0241 (7)	0.0004 (5)	0.0038 (5)	−0.0024 (6)
O4	0.0141 (6)	0.0162 (7)	0.0212 (7)	−0.0011 (5)	0.0035 (5)	−0.0003 (5)
O5	0.0259 (8)	0.0447 (11)	0.0394 (10)	0.0106 (7)	−0.0015 (7)	−0.0184 (8)
O6	0.0297 (8)	0.0375 (10)	0.0567 (11)	−0.0091 (7)	0.0201 (8)	0.0063 (8)
O7	0.0580 (11)	0.0372 (10)	0.0165 (8)	−0.0249 (8)	−0.0030 (7)	−0.0033 (7)
O8	0.0446 (12)	0.0181 (10)	0.0132 (9)	−0.0015 (8)	−0.0033 (8)	−0.0001 (7)
O8B	0.015 (3)	0.034 (4)	0.018 (3)	−0.002 (2)	0.000 (2)	0.006 (3)
O9	0.0179 (7)	0.0133 (7)	0.0293 (8)	0.0001 (5)	−0.0048 (6)	−0.0007 (6)
O10	0.0360 (9)	0.0361 (10)	0.0407 (10)	−0.0002 (8)	0.0079 (8)	−0.0205 (8)
C1	0.0140 (8)	0.0186 (9)	0.0128 (9)	0.0027 (7)	0.0003 (6)	0.0009 (7)
C2	0.0121 (8)	0.0210 (10)	0.0168 (9)	0.0015 (7)	−0.0004 (7)	−0.0032 (8)
C3	0.0188 (9)	0.0216 (10)	0.0245 (10)	−0.0022 (7)	0.0038 (8)	−0.0072 (8)
C4	0.0173 (9)	0.0334 (12)	0.0284 (11)	−0.0064 (8)	0.0063 (8)	−0.0119 (9)
C5	0.0141 (8)	0.0359 (12)	0.0264 (11)	0.0018 (8)	0.0000 (8)	−0.0150 (9)
C6	0.0208 (9)	0.0243 (11)	0.0322 (12)	0.0058 (8)	0.0002 (8)	−0.0085 (9)
C7	0.0173 (9)	0.0220 (10)	0.0275 (11)	0.0023 (7)	0.0022 (8)	−0.0020 (9)
C8	0.0186 (10)	0.0434 (14)	0.0350 (13)	0.0030 (9)	0.0016 (9)	−0.0222 (11)
C9	0.0131 (8)	0.0168 (9)	0.0127 (8)	−0.0013 (7)	0.0008 (6)	−0.0012 (7)
C10	0.0133 (8)	0.0175 (9)	0.0166 (9)	−0.0016 (7)	0.0004 (7)	0.0000 (7)
C11	0.0195 (9)	0.0186 (10)	0.0272 (11)	0.0000 (7)	0.0056 (8)	−0.0009 (8)
C12	0.0269 (10)	0.0175 (10)	0.0330 (12)	−0.0065 (8)	0.0047 (9)	−0.0012 (9)
C13	0.0189 (9)	0.0234 (10)	0.0263 (11)	−0.0038 (8)	0.0034 (8)	0.0052 (9)
C14	0.0159 (8)	0.0223 (10)	0.0269 (11)	0.0015 (7)	0.0055 (8)	0.0054 (8)
C15	0.0159 (8)	0.0178 (9)	0.0223 (10)	0.0005 (7)	0.0028 (7)	0.0032 (8)
C16	0.0291 (11)	0.0270 (12)	0.0439 (14)	−0.0085 (9)	0.0103 (10)	0.0046 (11)
C17	0.0200 (9)	0.0180 (9)	0.0156 (9)	−0.0029 (7)	0.0008 (7)	0.0023 (7)
C18	0.0252 (9)	0.0176 (10)	0.0168 (10)	−0.0064 (7)	−0.0022 (7)	0.0011 (8)
C19	0.0232 (9)	0.0173 (9)	0.0131 (9)	−0.0002 (7)	−0.0011 (7)	−0.0004 (7)
C20	0.0199 (9)	0.0226 (10)	0.0146 (9)	−0.0023 (7)	0.0015 (7)	0.0018 (8)
C21	0.0162 (8)	0.0197 (9)	0.0146 (9)	−0.0040 (7)	0.0006 (7)	−0.0004 (7)
C22	0.0354 (11)	0.0243 (11)	0.0148 (10)	−0.0056 (9)	−0.0033 (8)	−0.0001 (8)
C23	0.0188 (8)	0.0169 (9)	0.0147 (9)	0.0023 (7)	0.0008 (7)	−0.0023 (7)
C24	0.0210 (9)	0.0175 (9)	0.0176 (9)	0.0042 (7)	−0.0010 (7)	0.0013 (8)
C25	0.0179 (8)	0.0172 (9)	0.0133 (9)	−0.0029 (7)	−0.0009 (7)	0.0023 (7)
C26	0.0180 (9)	0.0217 (10)	0.0141 (9)	0.0015 (7)	0.0017 (7)	−0.0013 (7)
C27	0.0161 (8)	0.0191 (9)	0.0143 (9)	0.0022 (7)	0.0011 (7)	−0.0008 (7)
C28	0.0248 (9)	0.0204 (10)	0.0135 (9)	−0.0014 (8)	−0.0014 (7)	0.0031 (8)

### Geometric parameters (Å, °)

**Table:**

Cd1—O1	2.6055 (13)	C5—C6	1.392 (3)
Cd1—O2	2.3066 (13)	C5—C8	1.478 (3)
Cd1—O3	2.3368 (13)	C6—C7	1.387 (3)
Cd1—O4	2.5117 (13)	C6—H6	0.9300
Cd1—O9	2.3271 (15)	C7—H7	0.9300
Cd1—N1	2.3200 (16)	C8—H8	1.08 (3)
Cd1—N3	2.3362 (16)	C9—C10	1.505 (2)
N1—C17	1.342 (2)	C10—C11	1.388 (3)
N1—C21	1.337 (2)	C10—C15	1.393 (2)
N2—C22	1.324 (3)	C11—C12	1.390 (3)
N2—H2A	0.8600	C11—H11	0.9300
N2—H2B	0.8600	C12—C13	1.386 (3)
N3—C23	1.341 (2)	C12—H12	0.9300
N3—C27	1.343 (2)	C13—C14	1.389 (3)
N4—H4A	0.8600	C13—C16	1.479 (3)
N4—H4B	0.8600	C14—C15	1.383 (2)
N4B—C28	1.301 (7)	C14—H14	0.9300
N4B—H4B1	0.8600	C15—H15	0.9300
N4B—H4B2	0.8600	C16—H16	0.88 (3)
O1—C1	1.258 (2)	C17—C18	1.378 (3)
O2—C1	1.259 (2)	C17—H17	0.9300
O3—C9	1.255 (2)	C18—C19	1.393 (3)
O4—C9	1.263 (2)	C18—H18	0.9300
O5—C8	1.200 (3)	C19—C20	1.385 (3)
O6—C16	1.199 (3)	C19—C22	1.509 (3)
O7—C22	1.239 (3)	C20—C21	1.383 (3)
O8B—C28	1.263 (5)	C20—H20	0.9300
O9—H91	0.884 (15)	C21—H21	0.9300
O9—H92	0.875 (16)	C23—C24	1.381 (3)
O10—H101	0.880 (16)	C23—H23	0.9300
O10—H102	0.864 (18)	C24—C25	1.389 (3)
C1—C2	1.504 (2)	C24—H24	0.9300
C2—C7	1.391 (3)	C25—C26	1.386 (3)
C2—C3	1.392 (3)	C25—C28	1.507 (3)
C3—C4	1.389 (3)	C26—C27	1.386 (3)
C3—H3	0.9300	C26—H26	0.9300
C4—C5	1.381 (3)	C27—H27	0.9300
C4—H4	0.9300		
			
O2—Cd1—N1	100.95 (5)	O5—C8—C5	124.6 (2)
O2—Cd1—O9	137.10 (4)	O5—C8—H8	118.1 (15)
N1—Cd1—O9	82.70 (5)	C5—C8—H8	117.3 (15)
O2—Cd1—N3	88.61 (5)	O3—C9—O4	122.24 (16)
N1—Cd1—N3	165.76 (6)	O3—C9—C10	118.10 (16)
O9—Cd1—N3	83.11 (5)	O4—C9—C10	119.65 (15)
O2—Cd1—O3	82.61 (5)	C11—C10—C15	119.88 (17)
N1—Cd1—O3	91.08 (5)	C11—C10—C9	119.74 (16)
O9—Cd1—O3	140.29 (4)	C15—C10—C9	120.34 (16)
N3—Cd1—O3	100.70 (5)	C10—C11—C12	119.89 (18)
O2—Cd1—O4	134.07 (4)	C10—C11—H11	120.1
N1—Cd1—O4	94.69 (5)	C12—C11—H11	120.1
O9—Cd1—O4	87.35 (4)	C13—C12—C11	119.94 (19)
N3—Cd1—O4	85.95 (5)	C13—C12—H12	120.0
O3—Cd1—O4	53.96 (4)	C11—C12—H12	120.0
O2—Cd1—O1	52.91 (4)	C12—C13—C14	120.29 (18)
N1—Cd1—O1	85.68 (5)	C12—C13—C16	120.5 (2)
O9—Cd1—O1	85.26 (4)	C14—C13—C16	119.19 (19)
N3—Cd1—O1	91.86 (5)	C15—C14—C13	119.78 (18)
O3—Cd1—O1	133.54 (4)	C15—C14—H14	120.1
O4—Cd1—O1	172.50 (4)	C13—C14—H14	120.1
C21—N1—C17	117.70 (16)	C14—C15—C10	120.18 (18)
C21—N1—Cd1	120.40 (12)	C14—C15—H15	119.9
C17—N1—Cd1	121.32 (12)	C10—C15—H15	119.9
C22—N2—H2A	120.0	O6—C16—C13	124.5 (2)
C22—N2—H2B	120.0	O6—C16—H16	117.5 (18)
H2A—N2—H2B	120.0	C13—C16—H16	117.5 (18)
C23—N3—C27	117.90 (16)	N1—C17—C18	122.86 (17)
C23—N3—Cd1	119.37 (12)	N1—C17—H17	118.6
C27—N3—Cd1	122.27 (12)	C18—C17—H17	118.6
H4A—N4—H4B	120.0	C17—C18—C19	119.09 (17)
C28—N4B—H4B1	120.0	C17—C18—H18	120.5
C28—N4B—H4B2	120.0	C19—C18—H18	120.5
H4B1—N4B—H4B2	120.0	C20—C19—C18	118.26 (17)
C1—O1—Cd1	85.39 (10)	C20—C19—C22	123.69 (17)
C1—O2—Cd1	99.28 (11)	C18—C19—C22	118.01 (17)
C9—O3—Cd1	96.04 (11)	C21—C20—C19	118.83 (17)
C9—O4—Cd1	87.73 (10)	C21—C20—H20	120.6
Cd1—O9—H91	128.0 (16)	C19—C20—H20	120.6
Cd1—O9—H92	125.4 (16)	N1—C21—C20	123.19 (17)
H91—O9—H92	106 (2)	N1—C21—H21	118.4
H101—O10—H102	106 (3)	C20—C21—H21	118.4
O1—C1—O2	122.29 (16)	O7—C22—N2	123.34 (19)
O1—C1—C2	119.91 (16)	O7—C22—C19	118.77 (18)
O2—C1—C2	117.76 (16)	N2—C22—C19	117.89 (18)
C7—C2—C3	120.09 (17)	N3—C23—C24	122.90 (17)
C7—C2—C1	119.85 (17)	N3—C23—H23	118.5
C3—C2—C1	120.04 (17)	C24—C23—H23	118.5
C4—C3—C2	119.61 (19)	C23—C24—C25	119.00 (17)
C4—C3—H3	120.2	C23—C24—H24	120.5
C2—C3—H3	120.2	C25—C24—H24	120.5
C5—C4—C3	120.26 (19)	C26—C25—C24	118.43 (17)
C5—C4—H4	119.9	C26—C25—C28	119.09 (17)
C3—C4—H4	119.9	C24—C25—C28	122.48 (17)
C4—C5—C6	120.29 (18)	C25—C26—C27	119.04 (17)
C4—C5—C8	117.8 (2)	C25—C26—H26	120.5
C6—C5—C8	121.9 (2)	C27—C26—H26	120.5
C7—C6—C5	119.7 (2)	N3—C27—C26	122.65 (17)
C7—C6—H6	120.2	N3—C27—H27	118.7
C5—C6—H6	120.2	C26—C27—H27	118.7
C6—C7—C2	120.04 (19)	O8B—C28—N4B	125.1 (4)
C6—C7—H7	120.0	O8B—C28—C25	118.3 (3)
C2—C7—H7	120.0	N4B—C28—C25	116.6 (3)
			
O2—Cd1—N1—C21	95.74 (14)	C3—C4—C5—C6	−0.5 (3)
O9—Cd1—N1—C21	−127.58 (14)	C3—C4—C5—C8	−179.42 (19)
N3—Cd1—N1—C21	−132.88 (19)	C4—C5—C6—C7	−0.2 (3)
O3—Cd1—N1—C21	13.06 (14)	C8—C5—C6—C7	178.7 (2)
O4—Cd1—N1—C21	−40.86 (14)	C5—C6—C7—C2	1.1 (3)
O1—Cd1—N1—C21	146.65 (14)	C3—C2—C7—C6	−1.3 (3)
O2—Cd1—N1—C17	−93.18 (14)	C1—C2—C7—C6	−179.81 (18)
O9—Cd1—N1—C17	43.49 (14)	C4—C5—C8—O5	178.0 (2)
N3—Cd1—N1—C17	38.2 (3)	C6—C5—C8—O5	−0.9 (4)
O3—Cd1—N1—C17	−175.86 (14)	Cd1—O3—C9—O4	−1.43 (19)
O4—Cd1—N1—C17	130.21 (14)	Cd1—O3—C9—C10	177.44 (14)
O1—Cd1—N1—C17	−42.27 (14)	Cd1—O4—C9—O3	1.33 (18)
O2—Cd1—N3—C23	179.10 (14)	Cd1—O4—C9—C10	−177.53 (15)
N1—Cd1—N3—C23	46.6 (2)	O3—C9—C10—C11	15.4 (3)
O9—Cd1—N3—C23	41.28 (13)	O4—C9—C10—C11	−165.66 (18)
O3—Cd1—N3—C23	−98.69 (13)	O3—C9—C10—C15	−162.27 (18)
O4—Cd1—N3—C23	−46.53 (13)	O4—C9—C10—C15	16.6 (3)
O1—Cd1—N3—C23	126.28 (13)	C15—C10—C11—C12	−2.0 (3)
O2—Cd1—N3—C27	7.06 (14)	C9—C10—C11—C12	−179.72 (18)
N1—Cd1—N3—C27	−125.5 (2)	C10—C11—C12—C13	1.8 (3)
O9—Cd1—N3—C27	−130.76 (14)	C11—C12—C13—C14	0.0 (3)
O3—Cd1—N3—C27	89.27 (14)	C11—C12—C13—C16	178.3 (2)
O4—Cd1—N3—C27	141.42 (14)	C12—C13—C14—C15	−1.6 (3)
O1—Cd1—N3—C27	−45.76 (14)	C16—C13—C14—C15	−179.9 (2)
O2—Cd1—O1—C1	−1.99 (10)	C13—C14—C15—C10	1.3 (3)
N1—Cd1—O1—C1	−109.20 (11)	C11—C10—C15—C14	0.5 (3)
O9—Cd1—O1—C1	167.79 (11)	C9—C10—C15—C14	178.16 (18)
N3—Cd1—O1—C1	84.86 (11)	C12—C13—C16—O6	−176.7 (2)
O3—Cd1—O1—C1	−21.82 (13)	C14—C13—C16—O6	1.6 (4)
N1—Cd1—O2—C1	77.97 (12)	C21—N1—C17—C18	2.4 (3)
O9—Cd1—O2—C1	−13.06 (14)	Cd1—N1—C17—C18	−168.89 (15)
N3—Cd1—O2—C1	−91.39 (11)	N1—C17—C18—C19	−0.5 (3)
O3—Cd1—O2—C1	167.64 (12)	C17—C18—C19—C20	−1.6 (3)
O4—Cd1—O2—C1	−174.38 (10)	C17—C18—C19—C22	176.54 (18)
O1—Cd1—O2—C1	2.00 (10)	C18—C19—C20—C21	1.8 (3)
O2—Cd1—O3—C9	164.84 (12)	C22—C19—C20—C21	−176.23 (19)
N1—Cd1—O3—C9	−94.27 (11)	C17—N1—C21—C20	−2.2 (3)
O9—Cd1—O3—C9	−14.42 (14)	Cd1—N1—C21—C20	169.18 (15)
N3—Cd1—O3—C9	77.67 (11)	C19—C20—C21—N1	0.1 (3)
O4—Cd1—O3—C9	0.75 (10)	C20—C19—C22—O7	175.1 (2)
O1—Cd1—O3—C9	−179.32 (10)	C18—C19—C22—O7	−2.9 (3)
O2—Cd1—O4—C9	−22.99 (13)	C20—C19—C22—N2	−4.7 (3)
N1—Cd1—O4—C9	87.17 (11)	C18—C19—C22—N2	177.2 (2)
O9—Cd1—O4—C9	169.62 (11)	C27—N3—C23—C24	2.6 (3)
N3—Cd1—O4—C9	−107.10 (11)	Cd1—N3—C23—C24	−169.79 (14)
O3—Cd1—O4—C9	−0.74 (10)	N3—C23—C24—C25	−0.9 (3)
Cd1—O1—C1—O2	3.43 (17)	C23—C24—C25—C26	−1.7 (3)
Cd1—O1—C1—C2	−174.23 (16)	C23—C24—C25—C28	179.10 (17)
Cd1—O2—C1—O1	−3.9 (2)	C24—C25—C26—C27	2.4 (3)
Cd1—O2—C1—C2	173.79 (13)	C28—C25—C26—C27	−178.32 (17)
O1—C1—C2—C7	−164.72 (18)	C23—N3—C27—C26	−1.8 (3)
O2—C1—C2—C7	17.5 (3)	Cd1—N3—C27—C26	170.37 (14)
O1—C1—C2—C3	16.7 (3)	C25—C26—C27—N3	−0.7 (3)
O2—C1—C2—C3	−161.05 (18)	C26—C25—C28—O8B	−134.3 (4)
C7—C2—C3—C4	0.6 (3)	C24—C25—C28—O8B	44.9 (4)
C1—C2—C3—C4	179.12 (18)	C26—C25—C28—N4B	47.9 (5)
C2—C3—C4—C5	0.3 (3)	C24—C25—C28—N4B	−132.9 (4)

### Hydrogen-bond geometry (Å, °)

**Table:**

*D*—H···*A*	*D*—H	H···*A*	*D*···*A*	*D*—H···*A*
N2—H2A···O7^i^	0.86	2.04	2.891 (2)	172
N2—H2B···O10^ii^	0.86	2.05	2.888 (3)	165
N4—H4A···O8^iii^	0.86	2.01	2.863 (3)	174
N4—H4B···O5^iv^	0.86	2.01	2.852 (3)	166
N4—H4A···N4B^iii^	0.86	2.35	3.096 (7)	146
N4B—H4B1···N4^iii^	0.86	2.35	3.096 (7)	145
N4B—H4B1···O8B^iii^	0.86	2.02	2.880 (9)	178
N4B—H4B2···O7^v^	0.86	2.12	2.941 (7)	158
O9—H91···O4^vi^	0.88 (2)	1.86 (2)	2.740 (2)	175 (2)
O9—H92···O1^v^	0.88 (2)	1.87 (2)	2.740 (2)	174 (2)
O10—H101···O3	0.88 (2)	2.32 (3)	2.876 (2)	122 (2)
O10—H102···O2	0.86 (2)	1.90 (3)	2.766 (2)	175 (3)
C11—H11···O8^ii^	0.93	2.55	3.315 (3)	140
C17—H17···O1^v^	0.93	2.44	3.212 (2)	140
C20—H20···O10^ii^	0.93	2.35	3.258 (3)	166
C23—H23···O4^vi^	0.93	2.48	3.253 (2)	140
C26—H26···O6^vii^	0.93	2.55	3.137 (3)	122
C27—H27···O6^vii^	0.93	2.51	3.133 (3)	124

Symmetry codes: (i) −*x*, −*y*+1, −*z*−1; (ii) *x*, −*y*+3/2, *z*−1/2; (iii) −*x*+1, −*y*+1, −*z*+1; (iv) −*x*, *y*−1/2, −*z*+1/2; (v) −*x*, −*y*+1, −*z*; (vi) −*x*+1, −*y*+1, −*z*; (vii) *x*−1, −*y*+3/2, *z*+1/2.
